# Handheld NIR Spectroscopy Combined with a Hybrid LDA-SVM Model for Fast Classification of Retail Milk

**DOI:** 10.3390/foods13223577

**Published:** 2024-11-09

**Authors:** Francesco Maria Tangorra, Annalaura Lopez, Elena Ighina, Federica Bellagamba, Vittorio Maria Moretti

**Affiliations:** Department of Veterinary Medicine and Animal Sciences (DIVAS), Università degli Studi di Milano, Via dell’Università 6, 26900 Lodi, Italy; francesco.tangorra@unimi.it (F.M.T.); elena.ighina@unimi.it (E.I.); federica.bellagamba@unimi.it (F.B.); vittorio.moretti@unimi.it (V.M.M.)

**Keywords:** near-infrared spectroscopy (NIR), fatty acids, milk authentication, support vector machine

## Abstract

The EU market offers different types of milk, distinguished by origin, production method, processing technology, fat content, and other characteristics, which are often detailed on product labels. In this context, ensuring the authenticity of milk is crucial for maintaining standards and preventing fraud. Various food authenticity techniques have been employed to achieve this. Among them, near-infrared (NIR) spectroscopy is valued for its non-destructive and rapid analysis capabilities. This study evaluates the effectiveness of a miniaturized NIR device combined with support vector machine (SVM) algorithms and LDA feature selection to discriminate between four commercial milk types: high-quality fresh milk, milk labeled as mountain product, extended shelf-life milk, and TSG hay milk. The results indicate that NIR spectroscopy can effectively classify milk based on the type of milk, relying on different production systems and heat treatments (pasteurization). This capability was greater in distinguishing high-quality mountain and hay milk from the other types, while resulting in less successful class assignment for extended shelf-life milk. This study demonstrated the potential of portable NIR spectroscopy for real-time and cost-effective milk authentication at the retail level.

## 1. Introduction

The EU is the second largest milk producer globally, having delivered approximately 160 million tons of raw milk in 2022 [1]. Dairy production is a key pillar of the agricultural sector, representing more than 12% of total agricultural output in the EU [2]. In this context, Italy contributes to EU milk production with 14 million tons, accounting for 8.7% of total production, with 3000 processing plants employing 25,000 people [3].

A myriad of drinking milk types are commercialized in the EU, distinguished by their origin (i.e., geographical, species), production method (i.e., conventional, organic, mountain, hay milk), processing technology (i.e., raw, fresh, extended-life, ultra-filtrated, ultra-heated milk), fat content (i.e., whole, skimmed, or semi-skimmed milk), and other types (A2 milk, flavored milk, lactose-free milk) [4]. Such information is detailed on the labels, in compliance with EU food law or national legislation [5,6], but, in certain cases, these details may not be easily comprehensible or may be misunderstood by consumers.

This intricate spectrum of products has emphasized the importance of developing robust methods to ensure the authenticity of milk [7]. These methods contribute to verifying the adherence of the products to standards and regulations, preventing milk fraud and adulteration, and safeguarding the interests of producers and consumers.

Numerous food authenticity techniques have been extensively described in the recent literature [8,9,10]. Some of these techniques include spectroscopy, chromatography, isotopic and elemental techniques, nuclear magnetic resonance (NMR), sensory analysis, and molecular and immunological techniques. These enable the identification of specific indicators or characteristics unique to each food. Among these, fatty acids (FAs) composition, investigated by chromatographic analysis, has been effectively employed to discriminate different milk labels (such as mountain milk and hay milk from conventional milk) [11,12] at retail. However, the analytical protocol for FAs analysis, including fat extraction and quantification, FAs derivatization to their methyl esters (FAMEs), and chromatographic analysis (including calibration for FAs quantification), requires considerable time and resources. Which limits its practicality for routine use, especially in authenticity studies.

Conversely, near-infrared (NIR) spectroscopy is a powerful analytical technique widely used in food authentication due to its non-destructive nature, rapid analysis, environmental sustainability, and ability to provide detailed chemical information about food samples [13,14]. NIR spectroscopy is based on the principle of interaction between near-infrared light and the molecular vibrations of food components. When a sample is exposed to NIR radiation, certain wavelengths are absorbed by the sample’s chemical bonds, while others are transmitted or reflected. The resulting absorption spectrum contains valuable information about the molecular composition of the sample [15]. After data acquisition, different chemometric methods, including exploratory data analysis such as principal component analysis (PCA), regression (PLS), and classification methods (PLS-DA, LDA, SIMCA), are applied to interpret the complex spectral information and extract meaningful insights [16,17,18]. Chemometric models are developed using mathematical algorithms to correlate the spectral data with specific parameters of interest, such as moisture, fat, protein content, and food adulterants [19].

The increasing availability of portable NIRS technology has facilitated the exploration of several supervised techniques, including the support vector machine (SVM). Originally developed as a binary classifier, SVM has since been extended to handle multi-class problems through the use of kernel functions. Known for its simplicity and flexibility in classification, SVM is particularly valued for its computational efficiency and ability to achieve high performance compared to other machine learning classifiers [20]. However, a key challenge is the selection of the optimal kernel and its parameters, which can also be combined with data dimensionality reduction methods such as principal component analysis (PCA). PCA reduces data dimensionality by identifying principal components (PCs), which are orthogonal, uncorrelated, linear combinations of the original features that capture the maximum variance in the data [21]. PCA offers significant benefits, including improved generalization by minimizing noise and greater computational efficiency through feature reduction. This results in more robust models and faster training and prediction processes. By relying on PCs rather than raw spectra, the classifier gains accuracy. PCA-SVM classifiers have achieved high accuracy in distinguishing between different products such as honey [22], tea [23], and persimmons [24]. Recent studies have also demonstrated the effectiveness of PCA-SVM with Fourier-transform mid-infrared (FT-MIR) spectroscopy for non-destructive classification of milk based on geographical origin, achieving classification rates close to 95% [25]. Despite these advantages, PCA requires all the original features to derive the principal components, and these are often more difficult to interpret than the original data.

The classification accuracy of SVM can be improved using linear discriminant analysis (LDA), a linear classification method that maximizes the variance between groups and minimizes the variance within categories [26]. Because of its ability to reduce data dimensions, LDA facilitates data analysis and increases classification accuracy by better separating different classes [27]. Some studies aimed at determining the geographical origin of food have implemented the use of PCA prior to dimension reduction with LDA and classification with SVM [25,28,29].

In the dairy sector, NIR spectroscopy offers several advantages for authentication, including real-time analysis, minimal sample preparation, high throughput, and cost-effectiveness. It could be applied across various sectors of the dairy chain, including animal feed, raw milk production, milk processing, and cheese assessment, to verify the authenticity, quality, and safety of dairy products [30].

In recent years, the commercialization of several types of miniaturized NIR instruments [31,32,33] has brought significant advancements and new opportunities in the field of NIR application to milk authentication [30,34]. One of the primary advantages of miniaturized NIR instruments is their compact size and portability. They could be used for on-site analysis in various settings, like processing plants and field environments [35,36]. This portability facilitates real-time monitoring, rapid screening, and in situ measurements, offering greater flexibility for users [37]. In addition, they are more cost-effective if compared to traditional benchtop spectrometers. Indeed, their smaller size and simplified design enable manufacturers to offer these instruments at lower price points, making NIR spectroscopy more affordable and accessible to a broader range of users, including researchers and field technicians. On the other hand, they might have limitations in terms of performance compared to traditional benchtop spectrometers. Factors such as lower spectral resolution, reduced signal-to-noise ratio, and narrower spectral range may impact the accuracy, precision, and sensitivity of measurements, particularly for complex samples or analytes present at low concentrations [38].

In this study, we aimed to evaluate the effectiveness of a miniaturized NIR device combined with advanced chemometric techniques for the discrimination of four types of commercial milk: hay milk, fresh milk labeled as mountain product, high-quality fresh milk, and extended shelf-life milk. Our primary goal was to assess the device’s capability to accurately classify milk samples based on their characteristics. To achieve robust classification, we employed LDA and support vector machine (SVM) algorithms to analyze the data generated by the NIR device. This approach allowed us to determine the device’s discriminatory power and its potential utility for practical application in quality control and authenticity verification in the dairy industry.

## 2. Materials and Methods

### 2.1. Milk Samples

Eighty cartons or bottles of milk, depending on the producers, were purchased from several supermarkets in northern Italy. The milk was of four different types: high-quality fresh milk (HQ), mountain milk (MM), extended shelf-life milk (ESL), and hay milk (HM). Milk samples were collected on a weekly basis, spanning from March to September 2022, resulting in a total of 80 samples, 20 packs for each milk type. In pursuit of a comprehensive and representative selection, a variety of milk brands were included, guided by market availability during each respective sampling week. Only refrigerated full-fat milk samples with a shelf-life of fewer than 30 days were considered eligible for inclusion in this study, thereby focusing on products with a relatively short time frame from production to consumption.

This study encompassed four distinct types of retail milk, each contributing to the overall diversity of the dataset. The first type of milk was high-quality fresh milk (HQ) from intensive production systems. High-quality fresh milk derives from raw milk which underwent a single heat treatment of pasteurization (heating to approximately 72 °C for 15 s) within 48 h of milking to make it suitable for human consumption.

The second type was fresh milk labeled as mountain milk (MM). Mountain product is an optional quality designation established by Regulation (EU) No. 1151/2012 on quality schemes for agricultural products and foodstuffs [39]. The purpose of this designation is to enhance the marketing of mountain products and is exclusively used to identify products for which raw materials and animal feed essentially derive from mountain areas. The MM collected in this study was high-quality fresh milk, thus underwent a single heat treatment of pasteurization (approximately 72 °C for 15 s).

The third type of milk was extended shelf-life milk (ESL) from conventional production systems. ESL was pasteurized at high temperature for a short time (135 °C for 1–2 s). The shelf-life of ESL was around 20 days at refrigeration temperature.

The fourth type of milk was labeled as hay milk (HM). HM is a Traditional Specialty Guaranteed label (TSG) introduced by Regulation (EU) no. 2016/304 [40]. The HM is a dairy product of cows bred in traditional dairy farms and fed by grass, legumes, cereals, and hay without using fermented foods or supplying GMO feed. It is mandatory that, whatever the season, roughage make up at least 75% of the yearly ration of dry feed. Fermented fodder, such as silage, and fermented hay are strictly prohibited. The HM samples collected in this study were pasteurized at high temperature for a short time (135 °C for 1–2 s).

One-liter milk packages were collected and transported under refrigerated conditions to the university laboratories. Subsequently, approximately 60 mL of each milk type was transferred to sterile plastic tubes (4 × 15 mL each) and stored at −20 °C until analysis. The characteristics of the milk types used in this work are summarized in Table 1 along with data on fatty acid classes (saturated, monounsaturated, polyunsaturated, odd- and branched-chained) obtained on our previous investigation [41].

### 2.2. NIR Spectra Acquisition and Pre-Processing

An ultra-compact spectrometer, MicroNIR 1700 (VIAVI Solutions, Inc., San Jose, CA, USA), was used to collect the spectrometric data, connected to MicroNIR Pro 3.2 software. The device was equipped with the side view vial holder accessory that works in transflectance mode. This instrument is based on thin film linear variable filter (LVF) technology for the light dispersing element and works in the spectral region of 950–1650 nm, taking measurements at 125 spectral points spaced by approximately 6.2 nm. A white and a dark reference measurement was obtained using the dark-white reference assembly. The sensor integration time was 10 ms, and each spectrum was the mean of 3 scans.

Each milk sample was incubated at 37 °C for 30 min in a controlled-temperature bath (Grant Instruments, Royston, UK) before acquiring spectra. Three mL of each milk sample was placed into a 4 mL glass vial, and analyses were carried out in triplicate. The spectral data were exported in csv format for model development and statistical analysis. Triplicate readings were averaged for data analysis. Data were divided into training and validation sets with a ratio of 70:25, ensuring that each subset was representative of the overall distribution. Feature normalization was performed independently on the training and validation sets.

### 2.3. Classification Models

Classification models were built by combining LDA and SVM algorithms.

NIR spectral data were used as input to LDA, a classification technique commonly used in machine learning and pattern recognition. The linear method estimates the distance of each observation to the multivariate mean of each group using the Mahalanobis distance, and observations are classified into the closest group [42]. The LDA was performed using the discriminant platform of JMP Pro 17.2.

The SVM was trained on both the NIR data and the canonical scores identified by LDA. SVM is a supervised learning algorithm widely used to solve classification tasks. Based on the training data where the responses are known, the algorithm (kernel function) tries to find the optimal hyperplane, which can be used to classify new data points. The most commonly used kernels include linear and the Radial Basis Function (RBF) that create, respectively, a linear and a nonlinear hyperplane to separate the classes. The goal with a SVM is to fit a model such that the error between a predicted response and the actual response falls within a range of −ε to ε (insensitive region). The penalty associated with misclassifying an observation in the training set represents the cost parameter (C). A higher C parameter implements an algorithm that is less likely to misclassify a point in the training set, whereas a lower C parameter produces a wider margin. The SVM model’s performance depends on selecting the kernel and setting the parameters C and ε. Reducing ε usually increases the size of C [43]. In addition to the C parameter, the RBF kernel involves the definition of the gamma parameter (γ) that determines the amount of curvature there is to the decision line. A higher Gamma value indicates more curvature. Both the linear and the RBF kernels were tested using as minimum and maximum values for the parameters ε, C, and γ the JMP Pro 17 statistical software default values (ε = 0.1; C = 0.01 to 5; γ = 0.001 to 0.5) and repeating the procedure 20 times.

The performance of classification models was measured based on the highest accuracy rate calculated as the number of right classifications divided by the total number of observations. Multi-class confusion matrices were used for this purpose. To further evaluate the classification performance of the algorithms, the confusion matrices were converted into a one-vs-all type matrix (binary-class confusion matrix) to apply the following class-wise metrics based on the number of true positives (TP), false positives (FP), and false negatives (FN) from both dimensions of the matrix, with the remaining numbers contributing to true negatives (TN) [43] as follows:

Sensitivity (true positives over the count of actual positive outcomes):

(1) $$\frac{TP}{TP+FN}$$

Specificity (true negatives over the count of actual negative outcomes):

(2) $$\frac{TN}{TN+FP}$$

MCC (Matthews correlation coefficient):

(3) $$\frac{\left(TP\times TN\right)-\left(FP\times FN\right)}{\sqrt{\left(TP+FP\right)\times \left(TP+FN\right)\times \left(TN+FP\right)\times \left(TN+FN\right)}}$$

The range of this metric is from −1 to +1. In the case of an MCC of 1, we can assume that FP and FN are equal to 0, whereas in the case of an MCC of −1, we obtain a classifier that always misclassifies, hence we receive TP and TN equal to 0. As a rule of thumb, values of 0.70 or higher and −0.70 or lower indicate a very strong positive or negative relationship, respectively.

Statistical analysis and calibrations were performed using the statistical software JMP Pro 17.2 (SAS Institute, Cary, CA, USA) with the spectral tools developed by Worley [44].

## 3. Results and Discussion

In a previous study, we demonstrated that FAs profiling combined with statistical analysis can be a useful tool to distinguish the different types of retail milk considered in the study (HQ, MM, ESL, and HM). Data obtained through FAs analyses revealed significant differences among the four types of milk. Briefly, these showed notable differences for some FAs considered good markers of milk authenticity in terms of geographical origin (mountain or lowlands) and production system (organic, conventional, grass-based, or silage-based) [45,46], such as *trans*-C18:1 isomers, BCFAs, OCFAs, linoleic acid isomers, rumenic acid (CLA), and n-3 PUFAs. We detected higher concentrations for these FAs in HM, followed by MM, and then by HQ and ESL. In line with literature, these levels are generally higher in milk from cows fed with fresh herbage and roughage, as opposed to a corn silage-based diet [46]. We imputed these results to HM’s and MM’s product specifications, which prohibit the use of fermented feeds and mandate minimum 75% roughage in the annual dry matter cow ration (HM) and restrict the geographical origin of feedstuffs to mountain areas (MM), where dairy farming is mostly based on local forage production. On the contrary, we detected very few differences between HQ and ESL, which probably derived both from conventional production systems commonly spread in lowlands, where animals are mainly fed corn silage-based diets, characterized by lower forage-to-concentrate ratios.

Although the analytical protocol used to characterize the FAs profile of the different milk types provided interesting outcomes, it presents some limitations for routine application, particularly in authenticity studies, due to the extensive time and resources required. To address this, in this study, we explored NIR spectroscopy as a faster alternative and tested the discrimination ability of the four groups of milk based on fast NIR spectra acquisition combined with the use of machine learning techniques for classification.

Table 2 and Table 3 show the predicted and actual results of the observations, along with the overall accuracy, in the training and validation sets for the RBF kernel of NIR data and LDA-NIR data, respectively.

Generally, the RBF kernel of the SVM based on NIR data and LDA provided the highest overall accuracy for all milk types compared to the linear kernel. The combination of LDA and SVM improved the overall classification accuracy in the validation set (from 63.2% to 73.7%), as well as the classification accuracy between classes, compared to the NIR-based kernel. The accuracy in predicting MM, ESL, and HM in the validation set was the same (75%, 60%, and 80%, respectively), but the LDA-based SVM was once more accurate in predicting HQ samples (from 40% to 80% accuracy).

Interestingly, the LDA-SVM classification model applied to the NIR spectra of milk samples exhibited a certain percentage of misclassification among the milk classes. Notably, these misclassification errors primarily occurred between classes of milk that underwent the same thermal treatment (HQ and MM; ESL and HM). These outcomes suggest that the discrimination model based on NIR spectra and data analysis with variable selection based on LDA could be able to detect compositional differences in milk imparted by thermal treatment. Specifically, the HQ and MM classes were both subjected to standard pasteurization (72–80 °C/15–30 s), while the ESL and HM classes underwent high-temperature pasteurization (125–140 °C/1–10 s). This suggests that the model is sensitive to the changes induced by these different thermal processes, enabling a distinction in composition reflective of the treatment type, thus even HQ *vs* ESL. This outcome is very interesting since we were not able to detect any effect of the thermal treatment by analysis of fatty acids esterified in triacylglycerols of milk fat globules [41]. This could be imputed to thermal processing causing greater modification in other, more heat-sensitive milk components, such as whey proteins, vitamins, enzymes, and secondary compounds (i.e., furosine, hydroxymethylfurfural, and lactulose) [47,48]. We could hypothesize that these modifications altered the spectroscopic properties of milk, affecting molecular vibrations and thus generating different absorption spectra.

Since the combination of LDA-SVM showed greater accuracy, this combination was chosen to further investigate the power of the discriminant model. Table 4 shows the converted one-vs-all confusion matrices for each class of milk (HQ, ESL, HM, and MM) in the training and validation sets using SVM based on LDA-NIR data.

Table 5 shows the classification performance of the SVM RBF kernel based on the class-wise metrics (sensitivity, specificity, and MCC) on LDA-NIR data.

In the one-vs-all confusion matrix, it is observable that a good classification performance was achieved in discriminating HQ *vs* NOT HQ (80% sensitivity, 0.73 MCC), MM *vs* NOT MM (75% sensitivity, 0.68 MCC), and HM *vs* NOT HM (80% sensitivity, 0.62 MCC), while a lower performance in discriminating ESL *vs* NOT ESL (60% sensitivity, 0.57 MCC) was observed. These results seem to highlight a potential use of the portable NIR spectroscopy to discriminate different milk types with different labeling through fast in situ analysis.

The results from the classification of the different types of milk based on NIR obtained in this study align with previous studies describing the possibility of using NIRS to differentiate milk on the basis of the production system, mainly related to different diets administered to the cows, at farm level. Particularly, Martin et al. [49] and Noziere et al. [50] applied NIRS to bulk milk samples and differentiated between milk from grazing cows and cows fed corn silage and between milk from cows fed hay and cows fed grass. Valenti et al. [51] using a benchtop instrument (1100–2498 nm, reflectance mode) demonstrated the capability of NIRS to efficiently discriminate between milk samples from the hay and pasture-based systems and between milk samples from the corn silage and pasture-based systems, while a lower efficiency of discrimination was found for milk samples from corn silage and hay-based systems. The authors suggested spectroscopic methods (including MIR and NIR) as useful tools to discriminate different feeding systems in cow milk production chain at the farm level, with several parameters related to the farming system well known: geographical origin of milk, milk yield and composition, average breed composition for the herd, altitude of the farms, type of feedstuff (pasture, hay, grass silage, maize silage, or concentrate), and the estimated percentage of each feedstuff given to the herd. At the same time, Manuelian et al. [52] evaluated the feasibility of using visible/NIR spectroscopy (400–2500 nm, transmission mode) to discriminate organic from conventional bulk milk. However, authentication of organic bulk milk failed, likely due to the similarities in management and feeding of cows in the organic and conventional groups.

The abovementioned papers focused on spectroscopic data obtained using benchtop instruments on tank milk samples from farms. In the present study, we tested the ability of a portable NIR instrument to provide data (based on NIR spectra) suitable to detect differences and discriminate the considered milk labels at retail level. Actually, results from our discrimination model are based on data obtained from milk collected directly on the market. This milk originates from supply chains for which there is limited specific information available to authors, aside from the provisions stipulated by regulatory frameworks and the voluntary certification standards adhered to by these two categories of milk: Reg. EU 2016/304 for HM and Reg. EU 1151/2012 for MM. In this context, lacking specific information directly linked to farms and relying solely on commercial labels at the retail level, the ability to differentiate HM and MM using these techniques appears crucial for ensuring traceability and authenticity assurance.

Regarding the analytical technique based on portable tool, some authors previously compared models based on benchtop (1000–2500 nm, transmittance mode) and handheld NIRS (908–1676 nm, reflectance mode), showing equivalent ability to predict the identity of organic and conventional milk samples [34]. Some difficulties in classification were encountered when milk samples from pasture-fed cows were also included, as their patterns occasionally resembled those of the organic milk group. Furthermore, some authors utilized NIR data to predict FAs with the aim of discriminating milk samples from different production systems [45]. Notably, the n-3 PUFAs content of milk was predicted by NIR spectroscopy (1000–2500 nm, reflectance mode) to discriminate milk samples from organic and conventional production systems. A satisfactory prediction of α-linolenic acid and EPA (while no reasonable data were obtained using rumenic acid) was reached through NIR calibration equations, suggesting NIR as a fast and easy screening method to differentiate milk production systems (organic *vs* conventional), reducing the quantity of extensive and costly gas-chromatographic analysis necessary for FA profiling. In our research, the differentiation of different milk types was observed using both a classification model based on LDA-NIR data and the results obtained from chemical analysis of milk fat [41]. Interestingly, the latter suggested some individual FAs, particularly the *iso* form of some BCFAs, as interesting compounds to be proposed as additional markers (other than α-linolenic acid and EPA) to predict milk origin in HM and MM through NIR calibration equations and fast NIR analysis as a promising future perspective.

## 4. Conclusions

The appeal of milk associated with voluntary quality schemes has created a growing market share in recent years, but on the one hand the consumer cannot directly verify their authenticity, and on the other hand most of the methods used to guarantee the quality standard requirements are expensive and time-consuming. Therefore, a rapid, non-destructive, and inexpensive analysis to verify the conformity of the milk on the market with its label description represents a great opportunity. This study demonstrated the feasibility of using compact and portable NIR spectroscopy coupled with LDA-based SVM algorithms to rapidly classify different milk types without chemical pre-treatment at the retail level. This capability was greater for distinguishing high-quality milk, milk labeled as mountain product, and TSG hay milk, while resulting in less successful class assignment for extended shelf-life milk samples.

The practical application of this method lies in its ability to streamline quality verification in real-time, making it feasible for wider implementation in the supply chain, thereby enhancing consumer trust. The combination of LDA for initial data separation and SVM for precise classification allows for accurate milk classification even in complex scenarios, making it a valuable tool for quality assessment.

The use of a handheld NIRS enables on-site testing of milk samples at the retail level and potentially eliminates the need for laboratory analysis, significantly improving supply chain efficiency. The improved classification accuracy provided by the LDA-SVM approach enables retailers and quality control personnel to quickly identify adulterated or mislabeled milk, promoting food safety and consumer confidence as regular on-site testing can catch problems before products reach the consumer.

However, further studies are needed to investigate the possibility of employing the NIR technique, combined with appropriate calibration over specific clusters of fatty acids (primarily, the branched-chain FAs), to differentiate milk complying with voluntary quality schemes, including hay milk and mountain milk, focusing on a subset of significant compounds.

## Figures and Tables

**Table 1:** Characteristics of milk, as indicated on the label, and average composition of FAs of samples submitted to NIR analysis from previous published paper [41].

Label	High-Quality Fresh Milk (HQ)	Mountain Milk (MM)	Extended Shelf-Life Milk (ESL)	Hay Milk (HM)	
*N*	20	20	20	20	
**Heat treatment** °C/s	72–80 °C/15–30 s		125–140 °C/1–10 s		
**Average fat content** g/L	36	37	36	36	
**Average energy content** kCal/100 mL	65	66	65	66	
**Average price at retail** €/L	1.28	1.58	1.57	1.72	
**Fatty acids ^1^**					
mg 100 mL^−1^ of milk					*p* ^3^
SFAs ^2^	2060 ± 193.7	2202 ± 168.8	2067 ± 160	2120 ± 217.6	0.071
MUFAs ^2^	684 ± 59.0	708 ± 66.5	685 ± 47.1	698 ± 77.0	0.499
PUFAs ^2^	123 ± 13.8	127 ± 9.7	121 ± 11.0	137 ± 22.1	0.063
OCFAs ^2^	66 ± 6.7 ^b^	75 ± 7.1 ^a^	71 ± 7.0 ^b^	75 ± 9.0 ^a^	0.002 *
BCFAs ^2^	52 ± 4.6 ^d^	69 ± 7.3 ^b^	59 ± 6.4 ^c^	79 ± 8.0 ^a^	0.000 *

^1^ mean ± standard deviations. ^2^ SFAs: saturated fatty acids; MUFAs: monounsaturated fatty acids; PUFAs: polyunsaturated fatty acids; OCFAs: odd-chain fatty acids; BCFAs: branched-chain fatty acids. ^3^ An asterisk (*) and different superscripts in a row indicate significant differences (Kruskal–Wallis test, *p* < 0.05).

**Table 2:** Confusion matrix of milk type classification using SVM based on NIR data.

	Training Set (n = 55)						Validation Set (n = 19)				
	**Predicted**						**Predicted**				
**Actual**	*HQ*	*MM*	*ESL*	*HM*	**Overall Accuracy** 94.5%	**Actual**	*HQ*	*MM*	*ESL*	*HM*	**Overall Accuracy** 63.2%
*HQ*	13 (92.9%)	1	0	0		*HQ*	2 (40.0%)	2	0	1	
*MM*	1	11 (91.7%)	0	0		*MM*	1	3 (75.0%)	1	0	
*ESL*	0	0	14 (93.3%)	1		*ESL*	0	0	3 (60.0%)	2	
*HM*	0	0	0	14 (100%)		*HM*	0	0	1	4 (80.0%)	

HQ = high-quality fresh milk; MM = mountain milk; ESL = extended shelf-life milk; HM = hay milk; ( ) Accuracy in prediction for each milk class.

**Table 3:** Confusion matrix of milk type classification using SVM based on LDA-NIR.

	Training Set (n = 55)						Validation Set (n = 19)				
	**Predicted**						**Predicted**				
**Actual**	*HQ*	*MM*	*ESL*	*HM*	**Overall Accuracy** 81.8%	**Actual**	*HQ*	*MM*	*ESL*	*HM*	**Overall Accuracy** 73.7%
*HQ*	11 (78.6%)	1	1	1		*HQ*	4 (80.0%)	1	0	0	
*MM*	2	10 (83.3%)	0	0		*MM*	1	3 (75%)	0	0	
*ESL*	0	0	14 (93.3%)	1		*ESL*	0	0	3 (60.0%)	2	
*HM*	2	0	2	10 (71.4%)		*HM*	0	0	1	4 (80.0%)	

HQ = high-quality fresh milk; MM = mountain milk; ESL = extended shelf-life milk; HM = hay milk; ( ) Accuracy in prediction for each milk class.

**Table 4:** One-vs-all confusion matrices for each class of milk based on LDA-NIR data.

	Training Set (n = 55)			Validation Set (n = 19)	
	**Predicted**			**Predicted**	
**Actual**	*HQ*	*NOT HQ*	**Actual**	*HQ*	*NOT HQ*
*HQ*	11	3	*HQ*	4	1
*NOT HQ*	4	37	*NOT HQ*	1	13
	*MM*	*NOT MM*		*MM*	*NOT MM*
*MM*	10	2	*MM*	3	1
*NOT MM*	1	42	*NOT MM*	1	14
	*HM*	*NOT HM*		*HM*	*NOT HM*
*HM*	14	1	*HM*	3	2
*NOT HM*	3	37	*NOT HM*	1	13
	*ESL*	*NOT ESL*		*ESL*	*NOT ESL*
*ESL*	10	2	ESL	4	1
*NOT ESL*	4	39	NOT ESL	2	12

HQ = high-quality fresh milk; MM = mountain milk; ESL = extended shelf-life milk; HM = hay milk.

**Table 5:** Classification performance of the SVM based on LDA-NIR data for each milk class.

	Training Set (n = 55)			Validation Set (n = 19)			
Milk Class	Sensitivity (%)	Specificity (%)	MCC	Milk Class	Sensitivity (%)	Specificity (%)	MCC
*HQ*	78.6	90.2	0.67	*HQ*	80.0	92.9	0.73
*MM*	83.3	97.7	0.84	*MM*	75.0	93.3	0.68
*ESL*	93.3	92.5	0.83	*ESL*	60.0	92.9	0.57
*HM*	71.4	95.2	0.70	*HM*	80.0	85.7	0.62

HQ = high-quality fresh milk; MM = mountain milk; ESL = extended shelf-life milk; HM = hay milk.

## Data Availability

The original contributions presented in this study are included in the article. Further inquiries can be directed to the corresponding author.
